# Identification of a Benzenesulfonylpiperazine Derivative With In Vivo Efficacy Against Visceral Leishmaniasis

**DOI:** 10.1111/cbdd.70367

**Published:** 2026-08-04

**Authors:** Thibault Joseph William Jacques Dit Lapierre, Mariza Gabriela Faleiro de Moura Lodi Cruz, Guilherme Freitas de Lima Hercos, Rafael Consolin Chelucci, Simone Michelan‐Duarte, Gisele Barbosa, Analu R. Costa, Miguel Angel Chávez‐Fumagalli, Paula Derksen Macruz, Eduardo Jorge Pilau, Leonardo L. G. Ferreira, Adriano D. Andricopulo, Silvane Maria Fonseca Murta, Celso de Oliveira Rezende Júnior

**Affiliations:** ^1^ Laboratório de Síntese de Candidatos a Fármacos, Instituto de Química Universidade Federal de Uberlândia (UFU) Uberlândia MG Brazil; ^2^ Grupo de Genômica Funcional de Parasitos Instituto René Rachou, Fundação Oswaldo Cruz (FIOCRUZ Minas) Belo Horizonte MG Brazil; ^3^ Laboratório de Química Medicinal e Computacional (LQMC), Instituto de Física de São Carlos (IFSC) Universidade de São Paulo (USP) São Carlos SP Brazil; ^4^ Computational Biology and Chemistry Research Group, Vicerrectorado de Investigación Universidad Católica de Santa María Arequipa Peru; ^5^ Laboratório de Biomoléculas e Espectrometria de Massas (LaBioMass) Universidade Estadual de Maringá (UEM) Maringá PR Brazil; ^6^ Departamento de Química Universidade Federal de Juiz de Fora (UFJF) Juiz de Fora MG Brazil

**Keywords:** hit‐to‐lead, lead discovery, leishmaniasis, neglected tropical diseases, pharmacokinetics

## Abstract

Benzenesulfonylpiperazines have been previously identified as a promising class against Chagas disease and leishmaniasis, two parasitic neglected tropical diseases. Thus, the pharmacokinetic profile of two potential leads against visceral leishmaniasis was assessed in vitro. Both lead candidates **1** and **2** exhibited a satisfactory ADME profile, with **2** proving slightly superior in terms of metabolic stability. Therefore, after complementary toxicity assessment which revealed that **2** did not exert hepatotoxicity in vitro or acute toxicity in vivo, the evaluation of its efficacy in a mouse model highlighted that **2** indeed displays antileishmanial efficacy in vivo. Albeit moderate at a dose of 50 mg/kg/day, its efficacy increased at a dose of 100 mg/kg/day, reducing the parasite burden by 90% in the spleen of infected mice, though safety concerns were raised at this dose. In vitro investigation of its mode of action revealed that **2** exerts a cytostatic effect on *Leishmania infantum* promastigotes, promoting cell cycle arrest during the G0/G1 phase which may potentially be linked to the production of reactive oxygen species.

## Graphical abstract

This work highlights the satisfactory pharmacokinetic profile of two benzenesulfonylpiperazine lead candidates, followed by in vivo assessment in a murine model of visceral leishmaniasis. This assessment identified compound **2** as moderately active at a lower dose, but its efficacy increased at higher doses, though with concerns regarding safety. The mode of action of this class was also investigated in vitro.

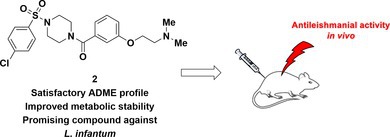

**Abbreviations**
ADME: absorption, distribution, metabolism and excretion
Ar atm.: argon atmosphere
CC_50_
: half maximal cytotoxic concentration
Cpd: compound
DCM: dichloromethane
DMF: *N,N*‐dimethylformamide
EDC: 1‐ethyl‐3‐(3‐dimethylaminopropyl)carbodiimide hydrochloride
eq: equivalent(s)
Et_3_N: triethylamine
HOBt: 1‐hydroxybenzotriazole
HPLC: high‐performance liquid chromatography
HRMS: high resolution mass spectrometry
IC_50_
: half maximal inhibitory concentration
*L. amazonensis*
: *Leishmania amazonensis*

*L. braziliensis*

: *Leishmania braziliensis*

*L. donovani*

: *Leishmania donovani*
*L. infantum*
: *Leishmania infantum*
MLM: mouse liver microsomes
MoA: mechanism of action
NMR: nuclear magnetic resonance
NOAEL: no‐observed‐adverse‐effect level
NTD: neglected tropical disease
ROS: reactive oxygen species
rt: room temperature
SI: selectivity index

1

Leishmaniases are parasitic diseases, currently classified as neglected tropical diseases (NTDs) by the World Health Organization and affecting millions of people worldwide (Alvar et al. 2012; Gebremichael Tedla et al. 2018). They are caused by a wide array of protozoans from the *Leishmania* genus, each parasite being associated with either one or both of the main forms of the disease, namely cutaneous and visceral leishmaniasis (Cecílio et al. 2022; Akhoundi et al. 2016). The latter is the most serious form of leishmaniasis, causing 20,000–40,000 deaths per year, despite the 50,000–90,000 new cases of visceral leishmaniasis being relatively low compared to those of cutaneous leishmaniasis, amounting to between 1 and 1.2 million (Gebremichael Tedla et al. 2018; van Griensven et al. 2024; Choi et al. 2021).

Among all *Leishmania* species, *Leishmania* (*Leishmania*) *donovani* ( *L. donovani* ) and *Leishmania* (*Leishmania*) *infantum* (*L. infantum*) account for most cases of visceral leishmaniasis. Geographical location is the main factor determining which species causes the infection, as *L. donovani* predominates in Asia and Africa, whereas *L. infantum* is more prevalent in the Mediterranean region, Middle‐East, Central Asia, and the Americas (Cecílio et al. 2022). *Leishmania* parasites have a digenetic life cycle involving two morphological forms that alternate between a *Phlebotominae* sand fly vector and a vertebrate host (Cecílio et al. 2022; Nagle et al. 2014). Thus, transmission of the disease is closely linked to the complex life cycle of these protozoans. Within the vector, parasites develop as extracellular promastigotes in the gut. During a blood meal, promastigotes are transmitted to the vertebrate host and internalized by phagocytic cells, where they differentiate into intracellular amastigotes. These amastigotes replicate within the host cells, resulting in a sustained infection and contributing to disease pathogenesis (Akhoundi et al. 2016; Nagle et al. 2014; Field et al. 2017).

Infection by *L. infantum* or *L. donovani* typically causes a prolonged fever, anemia and/or pancytopenia, a loss of body weight, and a localized darkening of the skin, and the migration of the parasite to the visceral organs results in splenomegaly and hepatomegaly as common symptoms of visceral leishmaniasis (Nagle et al. 2014; Burza et al. 2018). The lethality of the disease is mostly linked to the latter two symptoms and can rise up to 90% in the absence of appropriate treatment (Nagle et al. 2014; Burza et al. 2018).

In contrast with the pressing urgency for a prompt treatment of visceral leishmaniasis, viable therapeutic options currently consist of only a handful of drugs such as amphotericin B, miltefosine, or meglumine antimoniate (Figure 1A) (Choi et al. 2021; Majumder et al. 2023; Gupta et al. 2023). In addition to this unfortunate restricted number of options among approved antileishmanials, their high toxicity, high cost, variable efficacy, and the emergence of drug‐resistant *Leishmania* strains are among the several limitations faced by these compounds (Gupta et al. 2023; Singh et al. 2023; Sunyoto et al. 2019; Alves et al. 2018).

**FIGURE 1:**
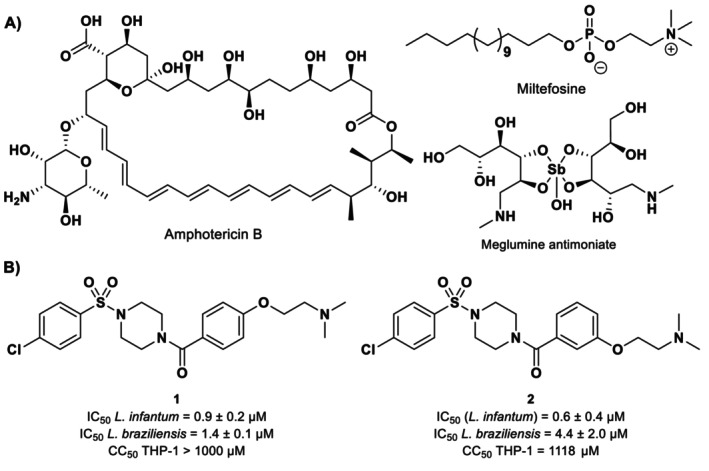
(A) Selected compounds used for the treatment of visceral leishmaniasis; (B) Structure and biological activity of lead candidates **1** and **2**.

Therefore, a significant number of drug discovery initiatives, aimed at overcoming the aforementioned limitations of current treatments with new compounds and scaffolds, were undertaken in recent years (Ferreira et al. 2021; Mishra et al. 2025; Thompson et al. 2018; Mowbray et al. 2021). One of such projects recently started with the study of the structure–activity relationship of the benzenesulfonylpiperazine scaffold against amastigotes forms of *Trypanosoma cruzi*, *L. infantum* and *L. braziliensis* , in which our team identified two derivatives with sub‐micromolar activity against *L. infantum* and high selectivity, which were therefore both deemed as potential candidates for in vivo assessment (compounds **1** and **2**, Figure 1B) (Freitas de Lima Hercos et al. 2024). Herein, the in vitro assessment of the pharmacokinetic profile of lead candidates **1** and **2** is reported, together with the investigation of the mechanism of action of **2** and the evaluation of its in vivo efficacy against *L. infantum* in a mice model of visceral leishmaniasis.

The synthesis of the in vivo candidates **1** and **2** was described in a previous study involving the benzenesulfonylpiperazine series (Freitas de Lima Hercos et al. 2024). Here, the same 3‐step route was followed for the scaled‐up synthesis of these two compounds, with some minor improvements over the previous, smaller‐scale synthesis (Scheme 1). Briefly, the common sulfonamide intermediate **3** was obtained from 4‐chlorobenzenesulfonyl chloride and a 3‐equivalent excess of piperazine, to favor the obtention of the mono‐sulfonamide over the possible di‐sulfonamide side product, in an improved 94% yield on a 2‐g scale. Then, amide coupling between **3** and the corresponding hydroxybenzoic acid mediated by EDC/HOBt provided **4** and **5** almost quantitatively, on a 500 mg scale. Such an improvement in terms of yield is quite noteworthy, especially for **5**, for which the previously reported yield was only 14% (Freitas de Lima Hercos et al. 2024). This sharp increase in yield for this step may be explained by the fact that no flash chromatography was required for the purification of **4** and **5**, therefore reducing potential losses of material over the column. Finally, the phenol moiety of both intermediates was alkylated with 2‐chloro‐*N,N*‐dimethan‐1‐amine hydrochloride to afford the in vivo candidates **1** and **2**. The alkylation of **4** into **1** was performed in the same conditions as previously described and led to a similar yield for the formation of **1**. However, replacing sodium hydride with sodium *tert*‐butoxide as the base for this S_N_2 reaction led to a near quantitative yield for the synthesis of **2** from **5**, much improved from the 67% previously described for this step. As a result, the scale‐up of the 3‐step route previously described proved successful for the synthesis of in vivo candidates **1** and **2**, affording the two derivatives in overall yields of 51% and 88%, respectively, greatly improved from the 38% and 8% overall yields previously reported (Freitas de Lima Hercos et al. 2024). Compounds **1** and **2** were characterized using NMR spectroscopy, HRMS, and HPLC analysis (Figures S1–S7).

**SCHEME 1:**
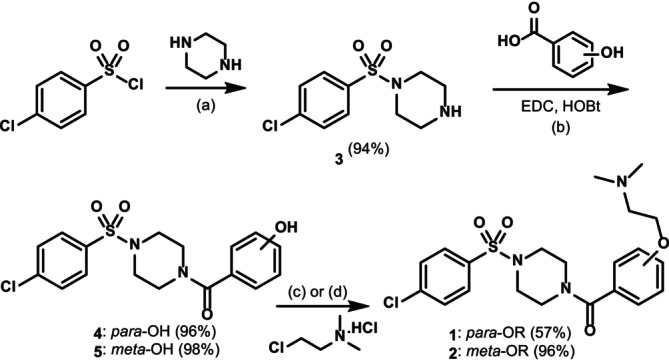
Synthesis of lead candidates **1** and **2**. Reagents and conditions: (a) Et3N, DCM, 0°C—rt, 14 h, 94%; (b) DMF, rt, 18–35 h, 96%–98%; (c) NaH (60% in mineral oil), DMF, Ar atm., 70°C, 24 h, 57% (for **1**); (d) tBuONa, DMF, 60°C, 48 h, 96% (for **2**).

To complement the antileishmanial profile of the series, compounds **1** and **2** were also tested against *L. amazonensis* amastigotes (Table 1). However, this species showed a greater resistance to benzenesulfonylpiperazines, as compounds **1** and **2** were roughly 8‐fold and 10‐fold less potent than against *L. infantum*, respectively.

**TABLE 1:** In vitro antileishmanial activities and ADME properties of lead candidates **1** and **2**.

Cpd	*L. infantum* EC_50_ (μM)^a^ (SI)^a^	*L. braziliensis* EC_50_ (μM)^a^ (SI)^a^	*L. amazonensis* EC_50_ (μM)^a^ (SI)^a^	elog(D) (pH 7.4)^b^	KS^c^		*P*_*e*_ (10^−6^ cm/s)^d^	MLM t_1/2_ (min)^e^	MLM Cl_int_ (μL/min/mg)^f^
					pH 2.0	pH 7.4			
**1**	0.9 ± 0.2 (> 1111)	1.4 ± 0.1 (> 714)	4.9 ± 0.5 (> 451)	2.90	87.1	99.3	1.78	69.31	40.00
**2**	0.6 ± 0.4 (1863)	4.4 ± 2.0 (255)	6.5 ± 0.3 (175)	2.97	73.3	79.5	2.38	88.87	31.20

^a^ EC_50_ value (μM) for the inhibition of the growth of amastigote forms of *L. infantum*, *L. braziliensis* , *and L. amazonensis* in THP‐1 macrophages (mean of at least two independent determinations, *p <* 0.05), SI: selectivity index (CC_50_/IC_50_).

^b^ Experimental lipophilicity, determined at pH = 7.4.

^c^ Kinetic solubility (μM).

^d^ PAMPA permeability. *P*_*e*_ < 1.5.10^−6^ cm/s: low permeability, *P*_*e*_ > 1.5.10^−6^ cm/s: high permeability.

^e^ Microsomal half‐life determined in Mouse Liver Microsomes (MLM), in presence of NADPH cofactor.

^f^ Intrinsic clearance determined in MLM, in presence of NADPH cofactor.

To select the most suitable of the two in vivo candidates for the assessment of its efficacy in a mice model of visceral leishmaniasis, the ADME profiles of **1** and **2** were determined in vitro. The pharmacokinetic properties considered in this profile included lipophilicity, water solubility, PAMPA permeability, and microsomal metabolic stability, to provide an accurate estimate of the bioavailability of **1** and **2** (Table 1). Overall, the two compounds displayed similar ADME profiles, which is to be expected given the similarity of their structures, only differing by the substitution pattern on the phenyl ring (**1**: *para* substituted, **2**: *meta* substituted). Both compounds were very similar in terms of lipophilicity and proved slightly lipophilic, though still followed Lipinski's rule of five (Lipinski et al. 1997) and remained within the recommended lipophilicity range (1 < elog(D) < 3) (Congreve et al. 2003; Leeson 2016). **1** and **2** also showed acceptable kinetic solubility at both acidic and neutral pH, well above their EC_50_ values, with **1** being the most soluble by a small margin. Interestingly, both compounds followed the same trend, proving slightly more soluble at a neutral pH than at an acidic one. Their PAMPA permeability also proved satisfactory, with both **1** and **2** deemed to readily permeate, though **2** exhibited a higher *P*_*e*_ than **1**, most probably due to being a slightly more lipophilic compound. Finally, **2** also performed better than **1** in terms of metabolic stability, exerting a microsomal half‐life 1.3 times longer than **1** and an intrinsic clearance lowered by the same factor, achieving so in spite of its higher lipophilicity.

Therefore, considering its satisfactory overall ADME profile and its slight edge over **1** in terms of metabolic stability and antileishmanial potency, **2** was ultimately prioritized for in vivo assessment of antileishmanial efficacy. This choice was further supported by its rather high CC_50_ value against HepG2 cells (CC_50_^HepG2^ = 59.27 μM), which suggests that **2** is not hepatotoxic. As later detailed, prioritizing **2** over **1** for in vivo studies proved justified, considering their outcome in terms of antileishmanial activity.

Concomitantly, its potential mode of action was also investigated in vitro. First, since this investigation was performed with a different *L. infantum* strain (MHOM/MA/67/ITMAP‐263) from the previous structure–activity relationship study, the activity of **2** against both the amastigote and promastigote forms of this strain had to be determined (Table 2). Then, flow cytometry was used to determine the effects of **2** and miltefosine (used as a reference compound) on the progression of the cell cycle in *L. infantum* promastigotes. Indeed, flow cytometry is a technique that allows the characterization of the effects of drugs and drug candidates on the cellular functions of unicellular organisms or cells, and was therefore used for the cell cycle assay. Promastigotes, and not clinically relevant intracellular amastigotes, were used since they provide a more experimentally accessible model for flow cytometry‐based mechanistic studies. This assay aims to identify the phase of the cell cycle in trypanosomatid parasites that is disrupted by drug candidates, by measuring the rate of DNA synthesis to determine whether the division process in cells has been interrupted. It also allows identifying whether an apoptotic or necrotic pathway led to the eventual death of the cells (Azas et al. 1997; Shapiro et al. 1979). Thus, *L. infantum* promastigotes were incubated with **2** and miltefosine at concentrations of 0.5 x EC_50_, EC_50_ and 2 × EC_50_ for 24 h. After treatment, parasites were collected, washed twice with PBS, and subjected to flow cytometry analysis. Initially, cell debris were excluded based on forward scatter (FSC) and side scatter (SSC) parameters. The promastigote population was selected according to FSC/SSC characteristics, and doublets were excluded prior to analysis. A minimum of 10,000 events was acquired per sample. For cell cycle analysis, parasites were stained with propidium iodide (PI), and DNA content histograms were used to determine the distribution of cells in the G0/G1, S, and G2/M phases. For apoptosis analysis, Annexin V‐FITC/PI staining was performed to distinguish between viable (Annexin C–/PI–), early apoptotic (Annexin V+/PI–), late apoptotic (Annexin V+/PI+), and necrotic (Annexin V–/PI+) cells. FITC and PI fluorescence were detected using the FL2 and FL3 (585 nm) channels, respectively. Data acquisition and analysis were performed using the Accuri C6 BD software. Results are representative of three independent experiments (Figure 2). This analysis showed a reduction in the number of cells, which occurred without triggering modifications in the cell cycle phases when compared to the untreated control. Cell cycle arrest was, however, noticeable during the G0/G1 phase, in which most cells were observed, which indicated an increase in the size of cellular organelles, which is characteristic of this phase. As most cells did not proceed to duplication of single‐copy organelles, cell cycle arrest was evident in the G0/G1 phase. Therefore, **2** was deemed to hinder the growth and proliferation of the parasite by halting the cell cycle in the G0/G1 phase, which further suggests that **2** may be cytostatic instead of cytocidal (Amlabu et al. 2020; Magalhães et al. 2022).

**TABLE 2:** In vitro antileishmanial activity of **2** against *L. infantum* amastigotes and promastigotes (MHOM/MA/67/ITMAP‐263 strain).

Cpd	*L. infantum* amastigotes EC_50_ (μM)^a^	*L. infantum* promastigotes EC_50_ (μM)^b^
**2**	3.50	0.57
**Miltefosine** ^**c**^	2.40	6.35

^a^ EC_50_ value (μM) for the inhibition of the growth of *L. infantum amastigotes* (*MHOM/MA/67/ITMAP‐263*) in THP‐1 macrophages (mean of at least two independent determinations, *p <* 0.05).

^b^ IC_50_ value (μM) for the inhibition of the growth of *L. infantum promastigotes* (*MHOM/MA/67/ITMAP‐263*) in THP‐1 macrophages (mean of at least two independent determinations, *p <* 0.05).

^c^ Miltefosine was used as a reference drug.

**FIGURE 2:**
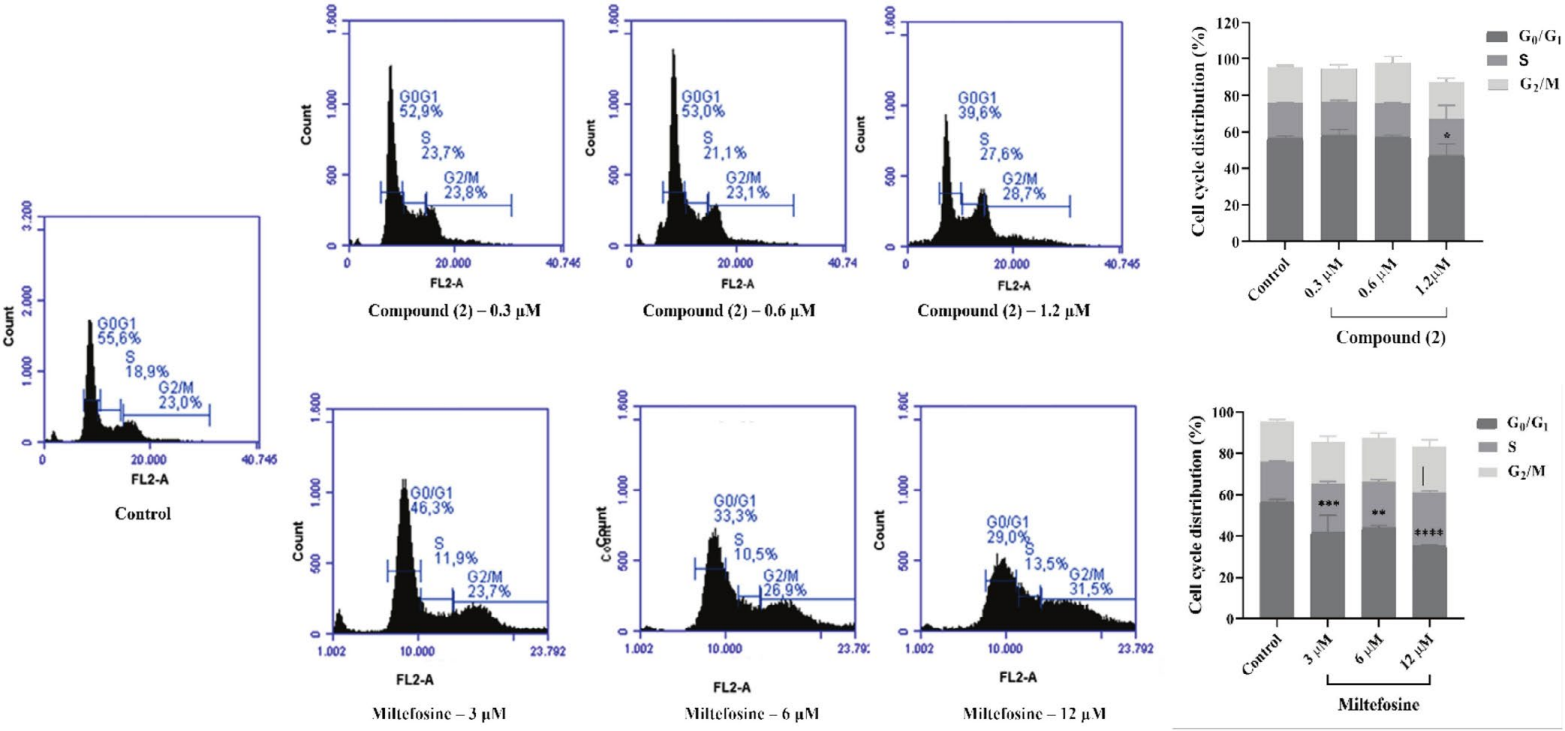
Flow cytometry histograms of *L. infantum* promastigote cells treated with **2** and miltefosine. Parasites were incubated with the compounds for 24 h. Cells of untreated promastigotes were used as a negative control. Percentages correspond to the quantification of cells at each phase of the cell cycle. Cell counts in treated wells were compared to those in untreated wells using Dunnet test, and statistical significance was set at *p < 0.01, **p < 0.003, ***p < 0.001, and ****p < 0.0001. Data shown is representative of three independent experiments.

This pattern of cell cycle arrest during the G0/G1 phase, and mainly during the G0 phase, is characteristic of apoptotic cells. Indeed, both early and late apoptosis could be observed among cells treated with **2** and miltefosine (Figure 3). It has been reported that the loss of cytoplasmic glutathione (GSH) could lead to apoptosis, as it alters the redox balance of cells and thus leaves them vulnerable to oxidizing agents (Slater et al. 1995). A decrease in mitochondrial GSH negatively affects the production of energy by the cell, which therefore undergoes late apoptosis and dies (Figure 3). Thus, a reactive oxygen species (ROS) assay was performed, as a mitochondrial dysfunction, caused by the alteration of the potential of the mitochondrial transmembrane and lower ATP levels, leads to the production of ROS, which in turn triggers late apoptosis (Jiang et al. 2017). As shown in Figure 4A, **2** induced a production of ROS in comparable amounts to H_2_O_2_ standards, thus suggesting that ROS production could be the pathway on which **2** acts to trigger apoptosis via cell cycle arrest during the G0/G1 phase and, ultimately, to exert its antiproliferative action on *L. infantum*.

**FIGURE 3:**
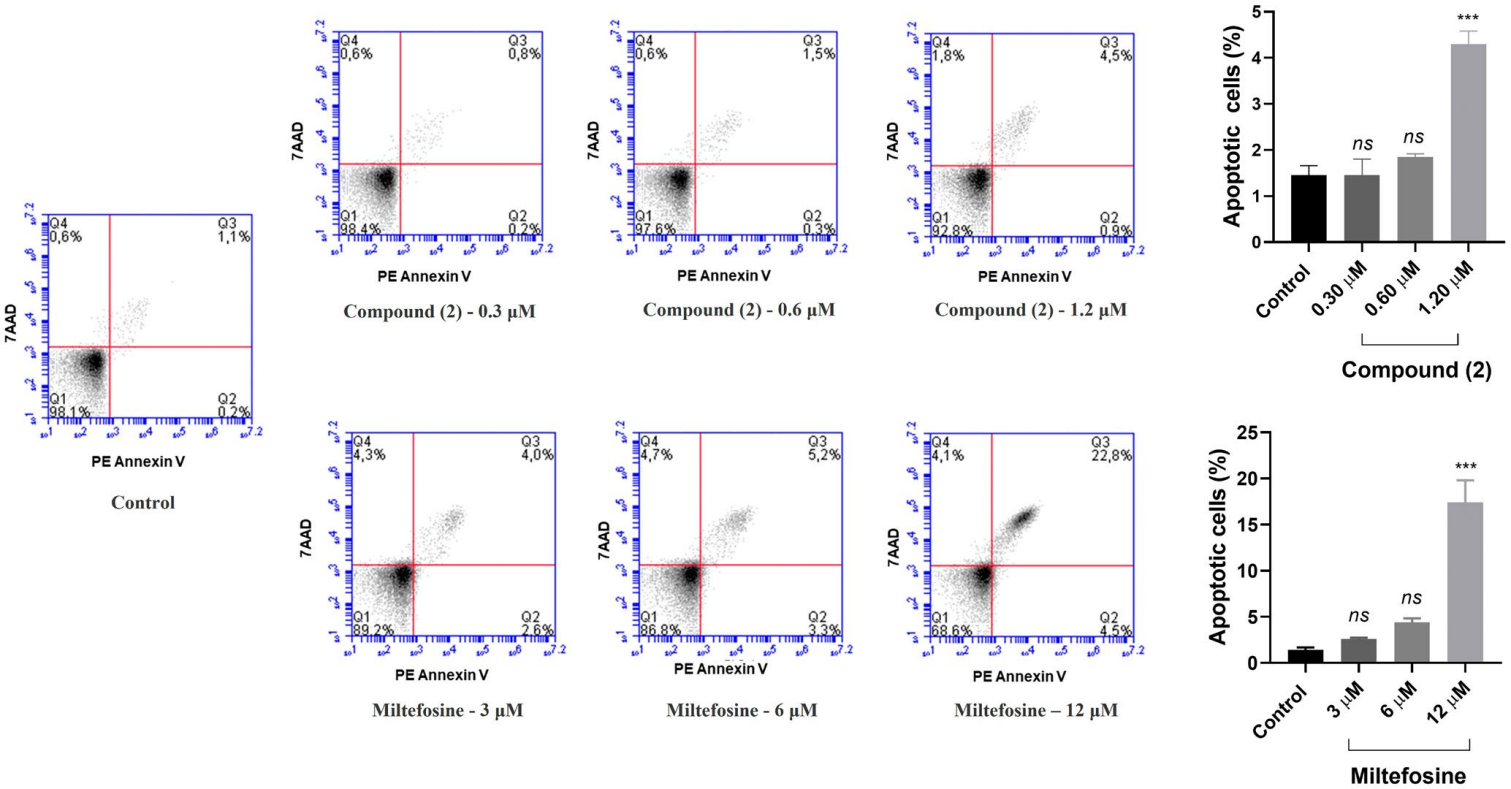
Flow cytometry histograms of **2** and miltefosine. Apoptotic rates were measured and analyzed by flow cytometry using Annexin V‐FITC/PI staining in *L. infantum*‐treated cells. Parasites were cultured with compounds for 24 h. Untreated promastigotes cells were used as negative control. Percentages correspond to the quantification of cells at each apoptotic phase. Cell counts in treated wells were compared to those in untreated wells using Dunnett test, and statistical significance was set at ***p < 0.001. Data shown is representative of three independent experiments.

**FIGURE 4:**
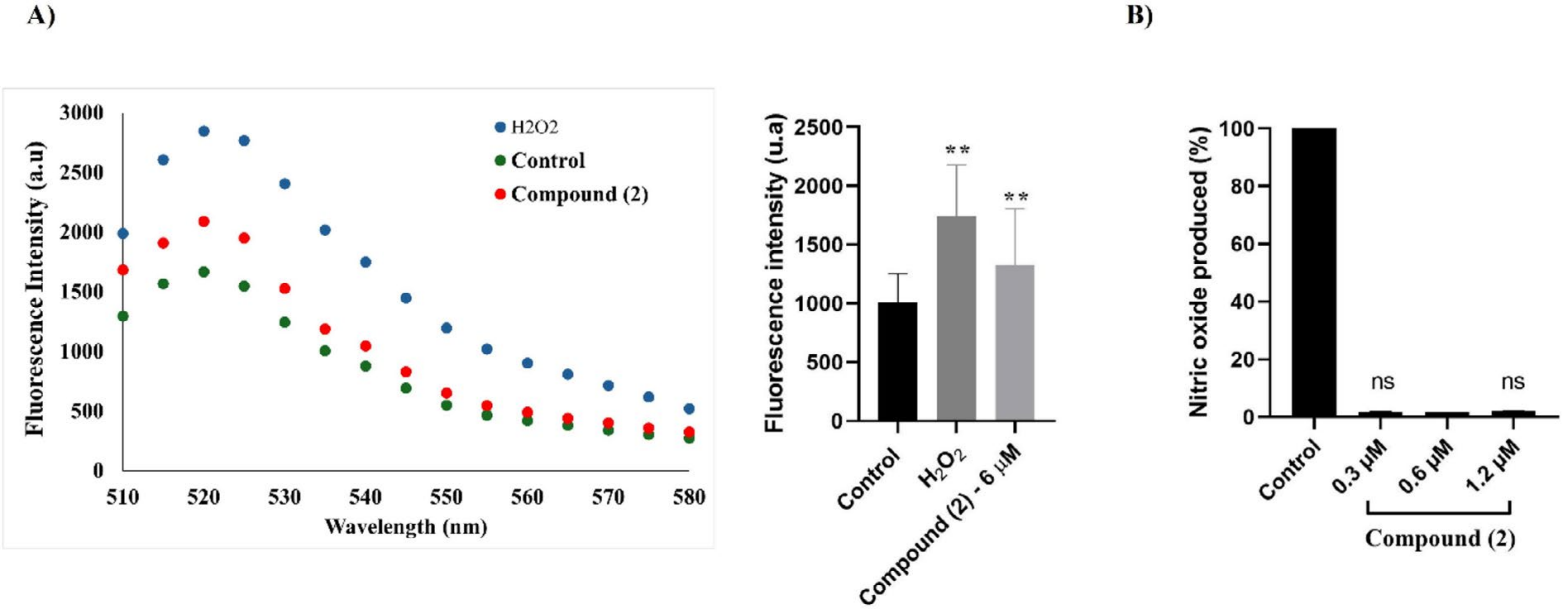
(A) Effect of **2** on reactive oxygen species (ROS) generation in *L. infantum* relative to the fluorescence generated. (B) Effect of **2** on nitric oxide (NO) production in *L. infantum* promastigotes. The statistical significance was set at **p < 0.001 and ns, non‐significant difference according to one‐way ANOVA. Data shown is representative of three independent experiments.

Then, since nitric oxide (NO) and ROS can be generated by either different enzymes or the same enzymes, alternating between reduction and oxidation processes in the latter case, a NO quantification assay was performed with **2** to confirm its hypothesized mechanism of action via ROS production. Satisfyingly, it did not induce any generation of NO in this assay (Figure 4B), thus suggesting that ROS production may contribute to the antiproliferative effects induced by **2** in *L. infantum* promastigotes, which consist in a cytostatic effect achieved by triggering cell cycle arrest during the G0/G1 phase. Nevertheless, additional mechanistic studies, including antioxidant rescue assays, will be required to establish whether parasite death is a direct result of the generation of ROS. Additionally, since these results were obtained with *L. infantum* promastigotes, they may not fully reflect the effects of **2** on amastigotes and thus remain preliminary insights into its mechanism of action, which are still to be confirmed in clinically relevant intracellular amastigotes.

The molecular structure of **2** was also analyzed using several bioinformatics methods (*in silico* target fishing) to screen possible molecular targets in *L. infantum* as well as the metabolic pathway. Using chemical similarity to identify proteins with known ligands, the SwissTarget, SEA, PharmMapper, and SuperPred servers revealed 100, 131, 290, and 127 human molecular targets, respectively, although only 72 proteins overlapped for more than two servers (Figure S8A). Upon analyzing the homologous sequences in the proteomes of *L. infantum* using the BLASTp program, 155 sequences were found to exhibit values beyond the cut‐off. Thereafter, the resulting sequences were uploaded to the Cytoscape platform for functional enrichment analysis (Figure S8B), where the top‐enriched term was GO:0036211, which represents the “protein modification process”. This term is associated with the covalent alteration of one or more amino acids occurring in proteins, peptides, and nascent polypeptides (EMBL‐EBI 2025). Within the STRING database, 588 proteins in *L. infantum* are associated with the GO:0036211 term, whereas upon analysis of the MCODE algorithm, eight main protein clusters were found, and the cytoHubba algorithm was used to calculate the centrality of proteins within the clusters (Figure S8C). The top central sequences were analyzed for the presence of druggable‐allosteric sites, with a cut‐off value of 50% of probability, and later docked against **2** (Figure S8D). The results showed that **2** bound, forming at least one H‐bond, to four molecular targets: the serine/threonine‐protein kinase TOR (Uniprot ID: A4IE36), the serine/threonine‐protein kinase TOR (Uniprot ID: A4IAK5), the non‐specific serine/threonine protein kinase (Uniprot ID: A4IAG3), and an uncharacterized protein (Uniprot ID: A4I1P7), which is annotated to have protein kinase activity (Figure S8E). Having identified these four proteins as potential targets, they should thus be prioritized in future mechanistic studies involving **2** and eventual optimized benzenesulfonylpiperazines, to confirm whether one of these is their actual molecular target in *L. infantum*.

Before assessing **2** in a murine model of visceral leishmaniasis, a complementary acute toxicity assay was performed in vivo, to identify potential adverse effects and determine its maximum tolerated dose. For this, increasing doses of **2** were administered intraperitoneally every 2 h to two BALB/c mice, up to a final accumulated dose of 100 mg/kg over 24 h. Inspection of the mice 24 h after the last administration of **2** did not identify any sign of acute toxicity in any animal, suggesting that this dose could be deemed safe for in vivo studies. Therefore, doses of 50 mg/kg and 100 mg/kg were used for the assessment of the antileishmanial efficacy of **2** in BALB/c mice.

To evaluate the efficacy of **2** in a murine model of visceral leishmaniasis, male BALB/c mice were infected with *L. infantum* parasites and randomly assigned to four different groups (6 mice per group). Two groups were treated with intraperitoneal injections of **2**, one at a dose of 50 mg/kg/day and the other at 100 mg/kg/day. The positive control group received meglumine antimoniate (500 mg/kg/day), and the control group was left untreated. All treatments lasted for 10 days and began 7 days after the initial infection. The efficacy of each treatment was assessed by determining the parasite burden in the spleen and liver of the treated mice relative to the control group, 3 days after completion of the treatment (20 days post‐infection) (Figure 5).

**FIGURE 5:**
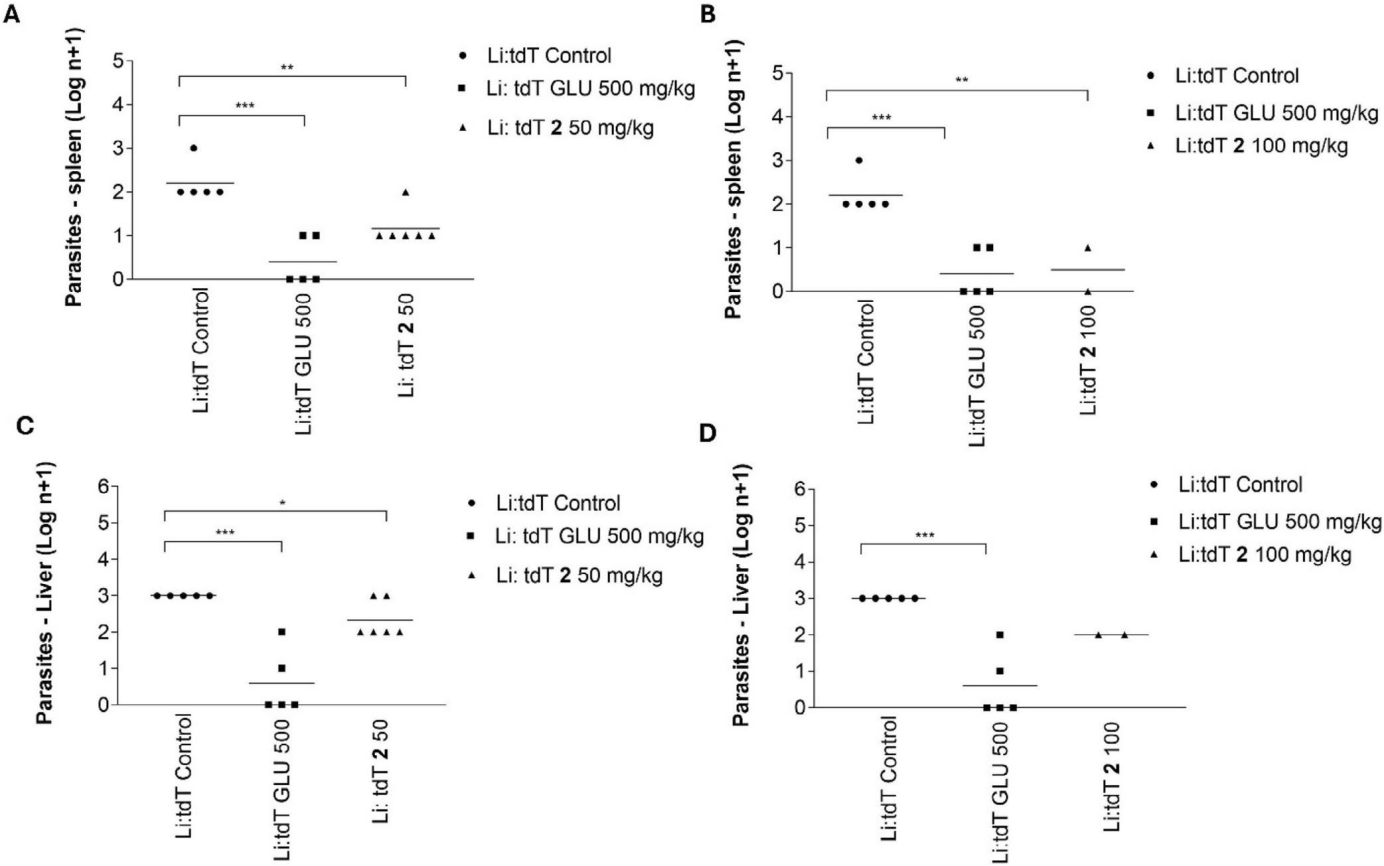
In vivo efficacy of **2** (50 mg/kg/day and 100 mg/kg/day) in a mice model of visceral leishmaniasis. Meglumine antimoniate (500 mg/kg/day) was used as a positive control, and a group was left untreated as the negative control. The antileishmanial efficacy was evaluated through the number of viable parasites recovered from the spleen (A and B) and the liver (C and D) of mice treated with **2** and meglumine antimoniate, when compared to the untreated control (a logarithmic scale was used for the graphical representation, [Log *n* + 1]). Groups receiving treatment were compared to the negative control using one‐way analysis of variance with Bonferroni *post hoc* test. Statistical analysis was performed using ANOVA followed by Bonferroni's multiple comparisons test (*p < 0.05; **p < 0.01; ***p < 0.001). GLU: Meglumine antimoniate. *Li:TdT*: Transfected *L. infantum::TdTomato* parasites.

Fortunately, no deaths nor clinical manifestations of acute toxicity were observed among the group treated with **2** at a daily dose of 50 mg/kg, as was also the case in both positive and negative control groups. However, such treatment only resulted in a reduction in parasite burden by 25% in the spleen of the animals, and by merely 10% in the liver. Increasing the dose to 100 mg/kg/day in **2** led to a sharp improvement in reducing the spleen parasite burden, which was decreased by 90% relative to the control group. Unfortunately, and despite this improvement regarding spleen parasite burden, four out of the six mice treated with **2** at a dose of 100 mg/kg/day showed signs of acute toxicity and died on the third or fourth day of treatment (10 and 11 days post‐infection, respectively), meaning that this 90% reduction in parasite burden was obtained from only two mice. Thus, both this 90% reduction in the spleen and the reduction in liver parasite burden among the two surviving animals were deemed not statistically significant.

As a result, despite the preliminary acute toxicity assessment indicating 100 mg/kg as a safe dose, the in vivo data shows that over a longer period of time, safety concerns begin to appear with **2**, as two‐thirds of the animals treated with this dose died. **2** proved nonetheless efficient at 100 mg/kg, since it achieved a 90% reduction in spleen parasite burden among the surviving mice, but the safety concerns at higher doses evidenced here by the death of four animals are too large to be outweighed by good efficacy. A lower dose in **2** appears to be safe, though at the cost of lower antileishmanial efficacy. The inconsistency between preliminary acute toxicity assessment and repeated‐dose in vivo treatment at 100 mg/kg suggests that the observed toxicity and mortality in mice may be related to cumulative exposure or drug bioaccumulation (Kip et al. 2018). Another hypothesis to explain the observed mortality could be that **2** exerts dose‐dependent chronic toxicity, with a chronic no‐observed‐adverse‐effect level (NOAEL) between 50 and 100 mg/kg/day, since no mortality was observed in a repeated‐dose treatment with the former dose while two‐thirds of animals died with the latter. Regardless, additional pharmacokinetic and toxicological characterization of **2** is needed in light of these results.

Thus, despite not being as efficient as what the sub‐micromolar activity in vitro could have suggested, the results from the treatment at a dose of 50 mg/kg/day remain a proof of concept of the in vivo antileishmanial efficacy of **2**, albeit low. As shown by the treatment at 100 mg/kg, increasing the dose could theoretically contribute to improving the efficacy, though this is in practice impossible due to the safety risks associated with a higher dose in **2**. Therefore, longer treatment periods with **2** at 50 mg/kg/day, or at a dose comprised between 50 and 100 mg/kg/day (ideally between 50 and 75 mg/kg/day), could arise as a viable alternative to increase the antileishmanial efficacy of **2** at lower doses while addressing the safety concerns at higher doses. Additionally, further optimization of this class may improve over the therapeutic profile of **2**, as structural modifications could enhance metabolic stability, increase selectivity towards its target, reduce off‐target toxicity, and improve the pharmacokinetic profile while maintaining the antileishmanial potency. Combining **2** with existing antileishmanial drugs may also represent a promising therapeutic strategy, potentially allowing a reduction in administered doses of **2** while maintaining or even improving efficacy, should future studies demonstrate synergistic or additive interactions. Such combination approaches could be especially valuable for minimizing the toxicity associated with prolonged treatment regimens, which remains a major challenge in antileishmanial chemotherapy. Finally, optimized drug delivery strategy, such as liposomal or polymeric nanoparticle encapsulation, could improve the in vivo safety and efficacy profile of **2**, as these could enhance selective accumulation within infected macrophages and reticuloendothelial tissues, increase bioavailability, prolong systemic circulation time, and reduce systemic exposure and peak‐related toxicity (Carneiro et al. 2010).

Taken together, the results presented herein highlight the potential of benzenesulfonylpiperazines as antileishmanial agents, and especially of **2** as the most promising representative of this class of compounds. Indeed, not only was its synthesis greatly improved from previous reports, but **2** also displayed a satisfactory pharmacokinetic profile in vitro for the current stage of development of its class against visceral leishmaniasis. Quite noteworthy, **2** also proved efficient against *L. infantum* promastigotes, in addition to its already known activity against the amastigote form, and in vitro investigation against the former form suggested that its mechanism of action is linked to a cytostatic effect, achieved by interrupting the cell cycle of *L. infantum* promastigotes during the G0/G1 phase and possibly associated with an increased production of ROS, though these mechanistic insights need to be confirmed in intracellular amastigotes. Ultimately, **2** exerted antileishmanial activity in vivo, effectively cementing its potential and, on a broader scope, the potential of the benzenesulfonylpiperazine class as a whole against visceral leishmaniasis. Albeit moderate at a daily dose of 50 mg/kg, the efficacy of **2** sharply increased at a dose of 100 mg/kg, although this increase came at the price of safety concerns associated with this higher dose. These concerns were in notable contrast with the results of the preliminary in vivo acute toxicity assessment, which had indicated 100 mg/kg as a safe dose for in vivo administration in mice, raising the hypothesis of a potential chronic toxicity displayed by **2** at higher doses, with an associated NOAEL between 50 and 100 mg/kg/day. In spite of these setbacks, **2** remains nonetheless a valuable compound within its class, acting as a proof of concept of the antileishmanial potential of benzenesulfonylpiperazines, and a viable starting point for future efforts aimed at identifying a safer benzenesulfonylpiperazine lead against visceral leishmaniasis.

## Author Contributions

**Mariza Gabriela Faleiro de Moura Lodi Cruz:** investigation, writing – original draft, methodology, writing – review and editing. **Thibault Joseph William Jacques Dit Lapierre:** investigation, methodology, formal analysis, writing – original draft. **Rafael Consolin Chelucci:** investigation, writing – review and editing, methodology. **Adriano D. Andricopulo:** funding acquisition, writing – review and editing, methodology, validation, supervision. **Paula Derksen Macruz:** investigation, methodology, writing – review and editing. **Miguel Angel Chávez‐Fumagalli:** investigation, methodology, writing – review and editing, validation. **Eduardo Jorge Pilau:** funding acquisition, methodology, validation, writing – review and editing, supervision. **Simone Michelan‐Duarte:** investigation, writing – review and editing, methodology. **Gisele Barbosa:** investigation, writing – review and editing, methodology. **Celso de Oliveira Rezende Júnior:** conceptualization, investigation, funding acquisition, writing – original draft, methodology, validation, visualization, formal analysis, project administration, supervision, resources. **Silvane Maria Fonseca Murta:** funding acquisition, writing – review and editing, methodology, validation, supervision. **Leonardo L. G. Ferreira:** funding acquisition, writing – review and editing, methodology, validation, supervision. **Analu R. Costa:** investigation, writing – review and editing, methodology. **Guilherme Freitas de Lima Hercos:** investigation, writing – review and editing, methodology.

## Funding

This work was supported by Fundação de Amparo à Pesquisa do Estado de Minas Gerais (RED‐00104‐22, RED‐00110‐23, APD‐01401‐25), Conselho Nacional de Desenvolvimento Científico e Tecnológico (309994‐2023‐3, 316892/2023‐8, 300079/2025‐7, 408678/2024‐0), Fundação de Amparo à Pesquisa do Estado de São Paulo (13/07600‐3, 24/12642‐1, 23/15953‐5).

## Conflicts of Interest

The authors declare no conflicts of interest.

## Supporting information


**Figure S1:** cbdd70367‐sup‐0001‐Figures.docx. ^1^H NMR spectrum of **1** (400 MHz, CDCl_3_).
**Figure S2:** ^13^C APT NMR spectrum of **1** (101 MHz, CDCl_3_).
**Figure S3:** ^1^H NMR spectrum of **2** (400 MHz, CDCl_3_).
**Figure S4:** ^13^C APT NMR spectrum of **2** (101 MHz, CDCl_3_).
**Figure S5:** HRMS (ESI +) spectrum of **1**.
**Figure S6:** HRMS (ESI +) spectrum of **2**.
**Figure S7:** HPLC traces of **2**.
**Figure S8:** *In silico* target fishing prediction of the molecular targets of **2**. Venn diagrams of the number of overlapping “hits” predicted by online servers (A). GO‐Biological process enriched terms are shown in a tree layout (B), while network diagrams of MCODE and CytoHubba analysis, where redder nodes indicate a higher degree of centrality (C). PASSer scores and binding affinity calculations for the selected molecular targets are shown in bar plots (D); the dotted line represents the cutoff value for the PASSer score (greater than 50%). The 2D representation of the interaction of **2** with those molecular targets that form H‐bonds is shown in (E).

## Data Availability

The data supporting the findings of this study are available within the article and its Support Information.
