# Supplementary material for: Identification of a Benzenesulfonylpiperazine Derivative With In Vivo Efficacy Against Visceral Leishmaniasis
Source: Chem Biol Drug Des. 2026 Aug 4;108(2):e70367. doi: 10.1111/cbdd.70367 (PMC13435819; doi:10.1111/cbdd.70367)
Supplement: Supplementary file 1 [file CBDD-108-e70367-s001.docx]

**Supporting Information for**

**Identification of a benzenesulfonylpiperazine derivative with *in vivo* efficacy against visceral leishmaniasis**

**Table of contents**

**1. Synthesis of compounds 1 and 2	S-2**

**2. NMR spectra of compounds 1 and 2	S-5**

**3. HRMS spectra of compounds 1 and 2	S-7**

**4. HPLC traces	S-8**

**5. Experimental procedures – *in vitro* ADME	S-9**

**6. Experimental procedures – mode of action of 2	S-11**

**7. Experimental procedures – *in vivo* experiments	S-13**

**8. *In silico* target-fishing	S-14**

# 1. Synthesis of compounds 1 and 2

*General information*

Unless specified, all reactions were performed under magnetic stirring, and commercial reagents and solvents were used without further purification. Dichloromethane (DCM), triethylamine (Et_3_N) and dimethylformamide (DMF) were dried with molecular sieves. Ethyl acetate (EtOAc) and hexane (Hex) were previously distilled. Flash column chromatography was performed using either Aldrich silica gel (35-70 *mesh*) or Macherey-Nagel silica gel (230-400 mesh ASTM). Analytical thin-layer chromatography (TLC) was performed on chromatography aluminum sheets impregnated with silica-gel 60 F_254_ (Sigma-Aldrich) and plate revelation was achieved using UV light (254 nm) and/or iodine atmosphere. All reactions were monitored by TLC. ^1^H, proton-decoupled ^13^C and ^13^C APT NMR spectra were acquired in CDCl_3_ or DMSO-*d_6_* at 400 MHz (^1^H) and 101 MHz (^13^C and ^13^C APT) (Bruker Ascend 400). Chemical shifts (δ) are reported in ppm using residual solvent peak as an internal standard (CDCl_3_: 7.26 ppm, DMSO-*d_6_*: 2.50 ppm, TMS: 0.00 ppm for ^1^H NMR spectra, and CDCl_3_: 77.16 ppm, DMSO-*d_6_*: 39.52 ppm for ^13^C and ^13^C APT NMR spectra). Peak multiplicity was reported using the following abbreviations: s = singlet, d = doublet, t = triplet, q = quartet, dd = doublet of doublets, m = multiplet. The multiplicity is followed by the coupling constant(s) in Hz and integration. Exchangeable protons were not observable on some NMR spectra. For ^13^C APT NMR spectra, peaks pointing upwards correspond to primary and tertiary carbons, and peaks pointing downwards correspond to secondary and quaternary carbons. High resolution mass spectrometry (HRMS) was measured using electrospray ionization (ESI) (Bruker Daltonics Corporation, Q-TOF geometry Impact II). The purity of **2** was confirmed to be > 95% by HPLC analysis.

*Synthesis*

**1-((4-chlorophenyl)sulfonyl)piperazine (3)**

A solution of 4-chlorobenzenesulfonyl chloride (2 g, 9.04 mmol) in DCM (20 mL) was added dropwise at 0 °C to a solution of piperazine (2.335 g, 27.12 mmol, 3 eq) and Et_3_N (958.8 mg, 9.48 mmol, 1.05 eq) in DCM (20 mL), and the reaction was stirred for 14 h at room temperature.

Water (30 mL) was added, and the aqueous layer was extracted with EtOAc (3 x 10 mL). The combined organic layers were dried over anhydrous Na_2_SO_4_, filtered, and the solvent was evaporated under reduced pressure to yield **3**, which was used without further purification.

**3**: white solid, 2.23 mg, 94%. **^1^H NMR (400 MHz, DMSO – *d_6_*)** *δ* 7.73 (s, 4H), 2.81 – 2.77 (m, 4H), 2.72 – 2.69 (m, 4H).

**(4-((4-chlorophenyl)sulfonyl)piperazin-1-yl)(4-hydroxyphenyl)methanone (4)**

4-hydroxybenzoic acid (291 mg, 2.11 mmol, 1.1 eq), EDC (440.9 mg, 2.30 mmol, 1.2 eq) and HOBt (31.1 mg, 0.23 mmol, 0.12 eq) were solubilized in DMF (4 mL), **3** (500 mg, 1.92 mmol) was added, and the reaction was stirred for 35 h at room temperature.

Cold water (20 mL) was added to quench the reaction, and the aqueous layer was extracted with EtOAc (2 x 20 mL). The combined organic layers were dried over anhydrous Na_2_SO_4_, filtered, and the solvent was evaporated under reduced pressure to yield **4**, which was used without further purification.

**4**: white solid, 711 mg, 96%. **^1^H NMR (400 MHz, DMSO – *d_6_*)** *δ* 7.68 (d, *J* = 8.7 Hz, 2H), 7.54 (d, *J* = 8.7 Hz, 2H), 7.26 (d, *J* = 8.7 Hz, 2H), 6.81 (d, *J* = 8.7 Hz, 2H), 3.72 (s, 4H), 3.03 (s, 4H). **^13^C NMR (400 MHz, DMSO – *d_6_*)** *δ* 171.2, 159.0, 138.8, 133.9, 128.8, 128.8, 128.6, 124.6, 114.3, 45.3.

**(4-((4-chlorophenyl)sulfonyl)piperazin-1-yl)(3-hydroxyphenyl)methanone (5)**

3-hydroxybenzoic acid (291 mg, 2.11 mmol, 1.1 eq), EDC (440.9 mg, 2.30 mmol, 1.2 eq) and HOBt (31.1 mg, 0.23 mmol, 0.12 eq) were solubilized in DMF (4 mL), **3** (500 mg, 1.92 mmol) was added, and the reaction was stirred for 18 h at room temperature.

Cold water (20 mL) was added to quench the reaction, and the aqueous layer was extracted with EtOAc (2 x 20 mL). The combined organic layers were dried over anhydrous Na_2_SO_4_, filtered, and the solvent was evaporated under reduced pressure to yield **4**, which was used without further purification.

**5**: white solid, 720 mg, 98%. **^1^H NMR (400 MHz, CDCl_3_)** *δ* 7.68 (d, *J* = 8.8 Hz, 2H), 7.54 (d, *J* = 8.8 Hz, 2H), 7.21 (td, *J* = 7.5, 1.2 Hz, 1H), 6.86 – 6.78 (m, 3H), 6.19 (s, 1H), 3.93 – 3.48 (m, 4H), 3.16 – 2.86 (m, 4H). **^13^C NMR (101 MHz, CDCl_3_)** *δ* 170.5, 156.5, 140.2, 135.9, 134.1, 130.1, 129.9, 129.2, 118.8, 117.8, 114.6, 45.5, 29.2.

**(4-((4-chlorophenyl)sulfonyl)piperazin-1-yl)(4-(2-(dimethylamino)ethoxy)phenyl)methanone (1)**

**4** (300 mg, 0.79 mmol) and NaH (60% dispersion in mineral oil) (189.05 mg, 7.88 mmol, 10 eq) were solubilized in anhydrous DMF (4 mL) under argon atmosphere, 2-chloro-*N*,*N*-dimethylethan-1-amine hydrochloride (226.9 mg, 1.58 mmol, 2 eq) was added, and the reaction was stirred for 24 h at room temperature under argon atmosphere.

Water (20 mL) was added, and the aqueous layer was extracted with EtOAc (3 x 10 mL). The combined organic layers were dried over anhydrous Na_2_SO_4_, filtered, and the solvent was evaporated under reduced pressure. The residue was then purified by flash column chromatography (EtOAc/Hex (6:4)) to yield **1**.

**1**: white solid, 203 mg, 57%. **^1^H NMR (400 MHz, CDCl_3_)** δ 7.68 (d, *J* = 8.6 Hz, 2H), 7.53 (d, *J* = 8.6 Hz, 2H), 7.30 (d, *J* = 8.7 Hz, 2H), 6.90 (d, *J* = 8.7 Hz, 2H), 4.07 (t, *J* = 5.7 Hz, 2H), 3.71 (s, 4H), 3.03 (s, 4H), 2.73 (t, *J* = 5.7 Hz, 2H), 2.33 (s, 6H). **^13^C NMR (101 MHz, CDCl_3_)** δ 170.60, 160.66, 140.07, 134.23, 129.80, 129.43, 129.25, 126.91, 114.64, 66.39, 58.30, 46.21, 46.05. **HRMS (ESI +):** calcd for C_21_H_27_ClN_3_O_4_S^+^ [M + H]^+^ = 452.1405 *m*/*z*, found [M + H]^+^ = 452.1410 *m*/*z*, error = 1.10 ppm.

**(4-((4-chlorophenyl)sulfonyl)piperazin-1-yl)(3-(2-(dimethylamino)ethoxy)phenyl)methanone (2)**

**5** (473 mg, 1.24 mmol) was solubilized in DMF (2.5 mL), 2-chloro-*N*,*N*-dimethylethan-1-amine hydrochloride (357.2 mg, 2.48 mmol, 2 eq) and ^t^BuONa (476.7 mg, 4.96 mmol, 4 eq) were added, and the reaction was stirred for 48 h at 60 °C.

Water (50 mL) was added, and the aqueous layer was extracted with EtOAc (3 x 15 mL). The combined organic layers were dried over Na_2_SO_4_, filtered, and the solvent was evaporated under reduced pressure to yield **4** as a brown solid.

**2**: beige/brown solid, 535.2 mg, 96%. **^1^H NMR (400 MHz, CDCl_3_)** *δ* 7.69 (d, *J* = 8.7 Hz, 2H), 7.54 (d, *J* = 8.7 Hz, 2H), 7.29 (d, *J* = 8.3 Hz, 1H), 6.99 – 6.94 (m, 1H), 6.88 – 6.85 (m, 2H), 4.06 (t, *J* = 5.6 Hz, 2H), 3.90 – 3.48 (m, 4H), 3.13 – 2.89 (m, 4H), 2.75 (t, *J* = 5.6 Hz, 2H), 2.35 (s, 6H). **^13^C NMR (101 MHz, CDCl_3_)** *δ* 170.3, 159.1, 140.1, 136.2, 134.1, 119.4, 116.5, 113.6, 66.2, 58.3, 45.9. **HRMS (ESI +):** calcd for C_21_H_27_ClN_3_O_4_S^+^ [M + H]^+^ = 452.1405 *m*/*z*, found [M + H]^+^ = 452.1404 *m*/*z*, error = 0.22 ppm. **Purity:** 96%

*High-resolution mass spectrometry (HRMS)*

High-resolution mass spectrometry (HRMS) was performed on a Q-TOF geometry Impact II spectrometer (Bruker Daltonics Corporation, Germany) equipped with an electrospray ionization source (ESI) and operated in positive ion mode. Parameters were set as follows for the analysis of each sample: capillary voltage: 4500 V, with an end plate offset potential of 500 V; nebulizer pressure: 0.4 bar; dry gas flow: 4 L/min; dry temperature: 180°C. Spectra were acquired with an acquisition rate of 1.00 Hz, monitoring a mass range from 50 to 700 *m/z*. A syringe pump (KDS Legato 100, KD Scientific, Holliston, MA, USA) was used at a flow rate of 10 µL/min to infuse the calibrant solution of sodium formate (10 mmol L^-1^; isopropanol:water; 1:1; v:v).

*Purity analysis by high-performance liquid chromatography (HPLC-UV-MS)*

High-performance liquid chromatography (HPLC) analysis was performed using a Shimadzu Prominence system (Shimadzu Corporation, Japan) equipped with a UV-Vis detector and coupled to an ion trap mass spectrometer (Bruker Amazon SL, Bruker Daltonics Corporation, Germany). The compound was solubilized in 500 µL of LC-MS grade methanol, and 10 µL were injected into the system. Chromatographic separation was carried out on an Eclipse Plus C18 column (150 × 4.6 mm, 3 µm particle size) using water (A) and methanol containing 0.1% formic acid (B) as the mobile phases under isocratic conditions (A:B, 30:70, v/v). The column temperature was maintained at 35 °C with a flow rate of 0.7 mL/min. Detection was performed at 254 nm. Mass spectrometric analysis was conducted using electrospray ionization (ESI) in positive mode. Data were processed using Bruker DataAnalysis 5.2 software. Purity was determined based on peak area percentage at 254 nm.

# 2. NMR spectra of compounds 1 and 2

Figure S1. ^1^H NMR spectrum of **1** (400 MHz, CDCl_3_)

Figure S2. ^13^C APT NMR spectrum of **1** (101 MHz, CDCl_3_)

Figure S3. ^1^H NMR spectrum of **2** (400 MHz, CDCl_3_)

Figure S4. ^13^C APT NMR spectrum of **2** (101 MHz, CDCl_3_)

# 3. HRMS spectra of compounds 1 and 2

Figure S5. HRMS (ESI +) spectrum of **1**

Figure S6. HRMS (ESI +) spectrum of **2**

# 4. HPLC traces

Figure S7. HPLC traces of **2**

# 5. Experimental procedures – *in vitro* ADME

All ADME analyses were performed using LC-MS/MS (liquid chromatography-tandem mass spectrometry). The LC-MS/MS system used consisted of a Prominence UFLC (Shimadzu Corporation, Kyoto, Japan) chromatography system, interfaced with an LCMS-8045 triple quadrupole mass spectrometer (Shimadzu Corporation, Kyoto, Japan) equipped with an electrospray ionization source (ESI).

*Experimental lipophilicity determination via distribution coefficient (log(D)_7.4_)*

For the determination of the distribution coefficient (elog(D)), a methodology based on the retention time of molecules in the stationary phase was used. The chromatogram was achieved on a Supelco Ascentis Express RP amide HPLC column (5 cm x 2.1 mm, 2.7 µM), using 5% methanol in 10 mM ammonium acetate pH 7.4 (A) and 100% methanol (B) as mobile phases. The mobile phase was eluted in binary gradient mode, as follows: 0 min: 95% A; 0.3 min: 100% A; 5.2 min: 0% A; 5.6 min: 0% A; 5.8 min: 100% A; 7.0 min: 100% A. The total run time was 7 minutes, and 5 µL of each sample were injected. The solutions of tested compounds were prepared at a concentration of 1.0 mg/mL in compound by adding the stock solution to a (1:1) mix of mobile phases A:B (internal standard at 200 nM), and the DMSO concentration was lower than 2%. The lipophilicity of compounds was assessed by individually injecting the test compounds and a series of eight commercial drugs, covering a log(D) range of -1.86 to 6.1 (acyclovir: -1.86, atenolol: 0.16, antipyrine: 0.38, fluconazole: 0.50, metoprolol: 1.88, ketoconazole: 3.83, tolnaftate: 5.40, and amiodarone: 6.10) (McGaw, 2010; Lombardo, 2002; Lombardo, 2004).The retention time (in minutes) of each of the eight standards was plotted against their log(D) values, and the resulting equation for the calibration curve (y = mx + b) was used to calculate the elog(D) values for the compounds from their retention time.

*Kinetic solubility*

To determine kinetic solubility, stock solutions of test compounds and controls (10 mM in DMSO) were transferred to two 96-well plates (incubation plate) in duplicates. PBS pH 7.4 or 2.0 (final concentration of 250 µM) was added to each well of the plates, and the DMSO concentration was kept lower than 2.5%. The plates were sealed and shaken for 24 hours (200 rpm/25 °C). The precipitates on the incubation plate were removed by centrifugation (15 min/3000 rpm/25 °C), and the supernatant fractions were quantified by LC-MS/MS. An intermediate standard solution diluted at 0.5 mM in acetonitrile:water (1:1) was prepared from a 10 mM standard solution. A calibration curve was prepared for each of the test compounds and controls by diluting several times the intermediate standard solution to reach the desired concentrations of 50, 40, 20, 2, and 1 µM. The resulting equation for the calibration curve (y = mx + b) was used to calculate the experimental concentration values for the compounds. The chromatogram for analysis was achieved on a Supelco Ascentis Express C18 column (3 cm x 2.1 mm, 5 µM), using water + 0.05% formic acid (A) and acetonitrile + 0.05% formic acid (B) as mobile phases. The mobile phase was eluted in binary gradient mode, and the gradient was as follows: 0 min: 98% A; 1.2 min: 2% A; 2.0 min: 2% A; re-equilibration time: 0.6 min, 98% A. The total run time was 2 minutes, and 5 µL of each sample were injected and eluted at a flow rate of 0.6 mL/min.

*Parallel artificial membrane permeability assay (PAMPA)*

The parallel artificial membrane permeability assay was carried out in a 96-well pre-coated PAMPA plate system (Corning Gentest # 353015). Solutions of the compounds were prepared by diluting the stock solutions (10 mM) in phosphate-buffered saline (PBS) pH 6.5 to a final concentration of 10 µM, then added to the donor portion of the plate (300 µL/well), while PBS pH 7.4 (200 µL/well) was added in the acceptor portion. The DMSO concentration in the solutions was lower than 1%. The two portions of the plate were then assembled, and the system was incubated (5 h/37 °C). Samples of the initial donor solution (T0) were collected and stored at -20 ºC. At the end of the incubation, samples were collected from the donor and acceptor plates, then added to plates containing a quenching solution (10% water and 90% methanol: acetonitrile (50:50) + 50 nM tolbutamide), and the T0 samples were treated similarly. The final concentrations of compounds in the donor, acceptor and T0 wells were quantified by LC-MS/MS. The chromatograms for analysis were achieved on a Supelco Ascentis Express C18 column (3 cm x 2.1 mm, 5 µM). The mobile phases consisted of water + 0.1% formic acid (A) and acetonitrile + 0.1% formic acid (B). The mobile phase was eluted in binary gradient mode, and the gradient was as follows: 0 min: 95% A; 0.05 min: 95% A; 0.3 min: 2% A; 0.7 min: 2% A; 0.8 min: 95% A; 1.15 min: 95% A; 2.0 min: 95% A. The total run time was 2 minutes, and 10 µL of each sample were injected and eluted at a flow rate of 0.7 mL/min. The results were used to calculate an effective permeability (*P_e_*) value. All PAMPA assays were performed in triplicates.

*Metabolic stability in Mouse Liver Microsomes (MLM)*

Metabolic stability was evaluated in pooled CD1 mouse liver microsomes (20 mg/mL, GIBCO). Solutions of the compounds were prepared at a concentration of 0.5 µM in compound and incubated with 0.25 mg/mL liver microsomes in phosphate-buffered saline, pH 7.4. The DMSO concentration in the solutions was lower than 1%. The reactions were started by adding 0.5 µM NADPH cofactor. Samples were taken at timepoints 0 (immediately following the addition of the cofactor), 5, 10, 20, 30, and 60 minutes. The reactions were stopped by adding a quenching solution containing acetonitrile:methanol (1:1) (internal standard at 50 nM). The samples were then centrifuged (3500 rpm/30 min) to obtain pellets of precipitated microsomal protein. The supernatant fractions were quantified by LC-MS/MS. Peak area ratios (analyte/internal standard) were converted to % remaining using the area ratio at time 0 as 100%. Half-life (t_1/2_ = ln(2)/k) in minutes and intrinsic clearance (Cl_int_ = k x 1000/(0.25)) in µL/min/mg were calculated using a non-linear regression from % remaining versus incubation time. From this plot, the slope (k) was determined. The chromatograms for analysis were achieved on a Supelco Ascentis Express C18 column (3 cm x 2.1 mm, 5 µM). The mobile phases consisted of water + 0.1% formic acid (A) and acetonitrile + 0.1% formic acid (B). The mobile phase was eluted in binary gradient mode, and the gradient was as follows: 0 min: 95% A; 0.05 min: 95% A; 0.3 min: 2% A; 0.7 min: 2% A; 0.8 min: 95% A; 1.15 min: 95% A; 2.0 min: 95% A. The total run time was 2 minutes, and 10 µL of each sample were injected and eluted at a flow rate of 0.7 mL/min. Microsomal stability tests were performed in triplicates.

*Hepatotoxicity*

HepG2 cells were seeded at 2 × 10^4^ cells/mL in DMEM, the plates were incubated for 24 h (5% CO_2_, 37 °C) and the compounds and doxorubicin (used as a positive control) were added in serial dilutions. After 72 h incubation, 15 µL of MTS reagent (CellTiter96®) were added to each well, and the plates were incubated for 4 h, then read using a microplate absorbance reader at 490 nm (Barltrop, 1991). Growth inhibition was expressed as a percentage of the absorbance of the negative control wells. The CC_50_ values were automatically calculated from the dose-response curves fitted using the log of the inhibitor concentration *vs* the normalized response between 0 and 100% with a variable slope.

# 6. Experimental procedures – mode of action of 2

*Antileishmanial Activity Assay*

THP-1 monocytes were plated at a density of 2 × 10⁴ cells per well in 100 μL of RPMI-1640 and differentiated with 20 ng/mL phorbol 12-myristate 13-acetate (PMA). Cells were incubated for 72 h at 37°C in a humidified atmosphere containing 5% CO₂. Subsequently, the culture medium was removed and late-stage *L. infantum* promastigotes (MHOM/MA/67/ITMAP-263) were added at 2 × 10⁵ parasites per well (100 μL). After 24 h to allow infection, non-internalized parasites were eliminated by medium replacement. Test compounds were then applied in ten-point serial dilutions ranging from 64 μM to 0.12 μM, and plates were further incubated for 72 h. Each experiment included untreated controls and miltefosine as a reference drug. At the end of the treatment period, cells were fixed with methanol and stained with Giemsa. The average number of intracellular amastigotes per THP-1-derived macrophage was quantified using an inverted microscope and manual cell counting. Antiparasitic activity was expressed as the percentage reduction in intracellular amastigote burden relative to untreated controls. EC₅₀ values were calculated from dose–response curves by plotting the logarithm of compound concentration against normalized response values (0–100%), as previously described (Tavares, 2018).

*Antileishmanial activity assay against promastigotes*

*L. infantum* promastigotes (MHOM/MA/67/ITMAP-263) were cultured in complete Schneider medium (SigmaAldrich, St. Louis, MO, USA), which consisted of Schneider medium plus 10% heat-inactivated fetal bovine serum (FBS, SigmaAldrich), pH 7.4, at 24 °C. The *L. infantum* promastigotes were plated at a concentration of 10^5^ parasites/mL, and **2** was added at concentrations of 64, 32, 16, 8, 4, 2, 1, 0.5, 0.25, and 0.125 µM. The cells were cultured for 72 h and, after the incubation time, cell proliferation was measured by colorimetric MTS assay. The cells in each well were stained with 20 µL of MTS (Promega, G3580) for 3 h, and the OD at 490 nm was determined with a SpectraMax 384 microplate reader (Sunnyvale, CA, USA) and the data was analyzed using GraphPad Prism® v.8.0. All experiments were independently repeated at least three times (Azzouz, 2017).

*Flow Cytometric Analysis of Sub G0/G1 of the Cell Cycle*

*L. infantum* promastigotes (MHOM/MA/67/ITMAP-263) at a concentration of 1×10^6^ parasites/mL were treated with the compounds for 24 h at concentrations of 0.5 x EC_50_, EC_50_, and 2 x EC_50_ (EC_50_ values determined against *L. infantum* promastigotes), while untreated parasites were used as a negative control and miltefosine as a reference standard. Then, the cells were collected, washed twice with 1 × PBS, and fixed in 70% ethanol at 4 °C for 24 h. The fixed parasites were then washed with 1 × PBS and the pellet was treated with 500 µL of RNase A (40 mg/mL) and 60 µg/mL propidium iodide (PI), and incubated at 25 °C for 1 h. Cell cycle distribution, determining the percentage of cells in G0, G1, S, and G2/M phases, was analyzed using an Accuri C5^TM^ flow cytometer (BD Biosciences) and data was analyzed using the BD Accuri C5^TM^ Samples software. The histograms presented are representative of results obtained from three independent experiments (Ali, 2021).

*FACS Analysis for Determination of Phosphatidylserine (PS) Externalization.*

An annexin V/7-amino-actinomycin D (7-AAD) apoptosis detection kit (BD Bioscience) was used for the detection of apoptotic or necrotic cell death in *L. infantum* promastigotes. After 24 h incubation with the compounds at concentrations of 0.5 x EC_50_, EC_50_ and 2 x EC_50_ (EC_50_ values determined against *L. infantum* promastigotes), *L. infantum* promastigotes (10^6^ cells/mL) were washed in phosphate-buffered saline (PBS) medium and centrifugated at 13000 rpm for 5 min, then stained with annexin V-PE/7-AAD for 15 min in the dark at 4 °C. Data was acquired and analyzed using an Accuri C5^TM^ flow cytometer (BD Biosciences) and the BD Accuri C5^TM^ Samples software, respectively (Aloui, 2016).

*Statistical Analysis*

All data is presented as Mean ± SD (standard deviation). Each experiment was conducted independently in triplicates. Statistical analysis was performed using an unpaired Dunnett test to assess significant differences between two groups, *p* < 0.05 was set as a significant level.

*Reactive Oxygen Species Production Assay*

The fluorescent dye H_2_DCFDA was employed to evaluate ROS production. *L. infantum* promastigotes (MHOM/MA/67/ITMAP-263) at a concentration of 1×10^6^ parasites/mL were treated either with **2** at a concentration equal to its EC_50_ against *L. infantum* promastigotes or with 100 µM H_2_O_2_ for 24 h. After treatment, the parasites were harvested, washed three times with 1 × PBS, and incubated with 20 µM H_2_DCFDA dye in 500 µM of 1 × PBS in the dark at room temperature for 30 minutes. Subsequently, the cells were centrifuged, resuspended in ice-cold PBS and kept at 0 °C until reading on a SpectraMax Gemini™ XPS/EM microplate reader. The median fluorescence intensity (MIF) was measured on a population of at least ten thousand cells and the data was further analyzed using Excel^®^ 2021 (Ali, 2021).

*Nitric Oxide Production Assay*

Nitric oxide production was quantified indirectly *via* nitrite production, using the Griess method (Green, 1982). THP-1 macrophages infected with *L. infantum* were treated with increasing concentrations of **2** for 24 h, and an aliquot (100 µL) of the supernatant was added with 100 µL of Griess reagent to a 96-well plate, in triplicates, and incubated for 10 min. The absorbance was then read at 540 nm on a SpectraMax Plus® microplate reader (Molecular Devices). Values were obtained from a standard calibration curve obtained with sodium nitrite.

# 7. Experimental procedures – *in vivo* experiments

All animal experiments were conducted in accordance with the guidelines and regulations of the Sociedade Brasileira de Ciência em Animais de Laboratório (SBCAL) and were approved by the Ethical Committee for Animal Experimentation of the Oswaldo Cruz Foundation – FIOCRUZ, under protocol number P52/22.2 (license no. LW-7/23). The study complies with the ACS Ethical Guidelines for the Publication of Chemical Research. BALB/c mice were obtained from the Centro de Bioterismo of the IRR/Fiocruz Minas. Free access to a standard diet was allowed during the whole study, and tap water was supplied *ad libitum*.

*Acute toxicity in vivo*

The maximum tolerated dose (MTD) of **2** was determined in BALB/c mice. One male and one female specimen, uninfected, six weeks old (ideal weight: 20 grams), were each administered intraperitoneally an emulsified sample prepared with 70% PBS1X, 20% PEG 400 (Polyethylene glycol 400, Sigma-Aldrich), 0.16% Tween 80 and 10% of **2**, with an initial dose of 5 mg/kg of **2**. After 2 h, an additional dose of 15 mg/kg was administered (resulting in an accumulated dose (AD) of 20 mg/kg). After another 2 h, a new dose of 30 mg/kg was administered (AD = 50 mg/kg). After a further 2 h, a final dose of 50 mg/kg (AD = 100 mg/kg) was administered. 24 h after the administration of the final dose, both mice were inspected for toxic and sub-toxic clinical manifestations according to Organization for Economic Co-operation and Development (OECD) rules (Walum, 1998; Mačianskienė, 2023). The inspection revealed no apparent signs of acute toxicity in both specimens.

*Antileishmanial efficacy in BALB/c mice*

BALB/c mice (male, 6 to 8 weeks old, 18 to 20 g) were inoculated intravenously (tail vein) with 2x10^7^ late-log-phase *L. infantum* promastigotes from *Li::tdTomato* lines, and randomly assigned into one of the four treatment groups (six mice per group). After 7 days of infection, each group was treated accordingly for 10 consecutive days: the positive control group was administered intraperitoneally in a single dose 500 mg/kg/day meglumine antimoniate (Glucantime®, Sanofi Medley), both groups treated with **2** received two daily intraperitoneal injections totaling either 50 mg/kg/day or 100 mg/kg/day of **2**, and the control group was administered the vehicle solution. For the intraperitoneal injection, **2** was formulated as follows: a stock solution of **2** in DMSO (100 mg/mL) was prepared daily, and a solution of 70% PBS1X, 20% PEG 400 (Polyethylene Glycol 400, Sigma-Aldrich), 0.16% Tween 80 and 10% of **2** (from the stock solution) was prepared. The control group was also administered this solution, replacing **2** with 10% DMSO. All mice were euthanized 3 days after the end of the treatment, and the liver and spleen of each animal were collected.

The number of viable parasites in each organ was determined using a quantitative limiting dilution assay. Briefly, the organs were macerated using an Ultra-Turrax disperser (IKA-Werke GmbH & Co. KG., Staufen, Germany), and a tissue homogenate was obtained with 1 mL of M199 medium. Each tissue homogenate was sequentially diluted (10-fold) in 96-well flat-bottom microtiter black plates and incubated at 26 °C for 10 days. The wells containing motile promastigotes were identified with an inverted microscope (Axiovert 25, Zeiss), and the parasite burden was determined from the highest dilution at which promastigotes had grown after 10 days of incubation.

# 8. *In silico* target-fishing

The chemical similarity principle-based target fishing approach used to screen for potential molecular targets of **2** was adapted from (Chávez-Fumagalli, 2018). Briefly, the Simplified Molecular Input Line Entry Specification (SMILES) of **2** was uploaded to the Superpred (https://prediction.charite.de/, accessed on 26 March 2025) (Gallo, 2022), SwissTargetPrediction (https://www.swisstargetprediction.ch/, accessed on 26 March 2025) (Gfeller, 2014), Similarity Ensemble Approach (SEA) (https://sea.bkslab.org/, accessed on 26 March 2025) (Lagunin, 2000), and PharmMapper (https://www.lilab-ecust.cn/pharmmapper/, accessed on 26 March 2025) (Liu, 2010) servers. Default parameters selected threshold values. A Venn diagram with the predicted molecular targets found by each server was generated with the “Calculate and draw custom Venn diagrams” web tool (http://bioinformatics.psb.ugent.be/webtools/Venn/). Since the main means offered by the servers to evaluate target fishing are related to human proteins, a cross-reference with *L. infantum* (taxid: 5671)-related proteins was performed using the BLASTp program (Boratyn, 2013), considering homologous sequences with an expected value (e-value) lower than 0.005 and a hit score larger than 100.0 (Collins, 1988). Afterward, the resulting sequences were uploaded to the Cytoscape platform (Shannon, 2003), where the plugin *“stringApp”* was used for functional enrichment (Doncheva, 2019). A tree layout was generated for visualization of the enrichment analysis within the yFiles Layout Algorithms (Wiese, 2004). Biological Process (BP) gene ontology (GO) protein–protein interaction (PPI) networks were retrieved from the Search Tool for the Retrieval of Interacting Genes/Proteins (STRING) database (v12.0) (https://string-db.org, accessed 30 April 2025) and imported into the Cytoscape platform, where the Molecular Complex Detection (MCODE) clustering algorithm plugin was used to detect connected regions in the networks that may represent molecular complexes (Bader, 2003), while network centrality measures were computed using the cytoHubba plugin (Chin, 2014) with node ranking based on the Maximal Clique Centrality (MCC) algorithm to identify key hub proteins. The molecular models of selected sequences were retrieved from the AlphaFold Protein Structure Database (Varadi, 2024) (accessed on 20 June 2025), while the PASSer (Protein Allosteric Sites Server) (https://passer.smu.edu, accessed 30 April 2025) (Tian, 2023) was employed to provide a table summarizing the predictions of the top pockets with the highest probabilities/scores of the allosteric sites, which are represented as the structures of druggable pockets. The molecular docking simulations were performed using the AutoDock Vina tool (Trott, 2010). The predicted molecular models were uploaded as macromolecules, and a thorough search was carried out by enabling the “Run AutoGrid” option, which creates configuration files for the grid parameter’s lowest energy pose. This was followed by the “Run AutoDock” option, which utilizes the Lamarckian GA docking algorithm. The docking simulation was then run with an exhaustiveness setting of 20 and instructed to produce only the lowest energy pose.

Figure S8. In silico target fishing prediction of the molecular targets of **2**. Venn diagrams of the number of overlapping “hits” predicted by online servers (A). GO-Biological process enriched terms are shown in a tree layout (B), while network diagrams of MCODE and CytoHubba analysis, where redder nodes indicate a higher degree of centrality (C). PASSer scores and binding affinity calculations for the selected molecular targets are shown in bar plots (D); the dotted line represents the cutoff value for the PASSer score (greater than 50%). The 2D representation of the interaction of **2** with those molecular targets that form H-bonds is shown in (E).
